# The reach of gene–culture coevolution in animals

**DOI:** 10.1038/s41467-019-10293-y

**Published:** 2019-06-03

**Authors:** Hal Whitehead, Kevin N. Laland, Luke Rendell, Rose Thorogood, Andrew Whiten

**Affiliations:** 1 Department of Biology, Dalhousie University, Halifax, B3H 4R2 Canada; 2 Centre for Social Learning and Cognitive Evolution, School of Biology, University of St Andrews, St Andrews, KY16 9TF United Kingdom; 3 Department of Zoology, University of Cambridge, Cambridge, CB2 3EJ United Kingdom; 4 Helsinki Institute of Life Science, University of Helsinki, Helsinki, 00014 Finland; 5 Faculty of Biological and Environmental Sciences (Research Program in Organismal & Evolutionary Biology), University of Helsinki, Helsinki, 00014 Finland; 6 Centre for Social Learning and Cognitive Evolution, School of Psychology and Neuroscience, University of St Andrews, St Andrews, KY16 9JP United Kingdom

**Keywords:** Evolutionary ecology, Coevolution, Cultural evolution, Evolutionary genetics

## Abstract

Culture (behaviour based on socially transmitted information) is present in diverse animal species, yet how it interacts with genetic evolution remains largely unexplored. Here, we review the evidence for gene–culture coevolution in animals, especially birds, cetaceans and primates. We describe how culture can relax or intensify selection under different circumstances, create new selection pressures by changing ecology or behaviour, and favour adaptations, including in other species. Finally, we illustrate how, through culturally mediated migration and assortative mating, culture can shape population genetic structure and diversity. This evidence suggests strongly that animal culture plays an important evolutionary role, and we encourage explicit analyses of gene–culture coevolution in nature.

## Abstract (web summary)

The reciprocal interaction between genetic and cultural evolution is well recognised in humans. Here, Whitehead and colleagues review the growing body of evidence that culture is also a major driver of both neutral and adaptive genetic evolution in non-human animals.

## Introduction

**Fig. 1:**
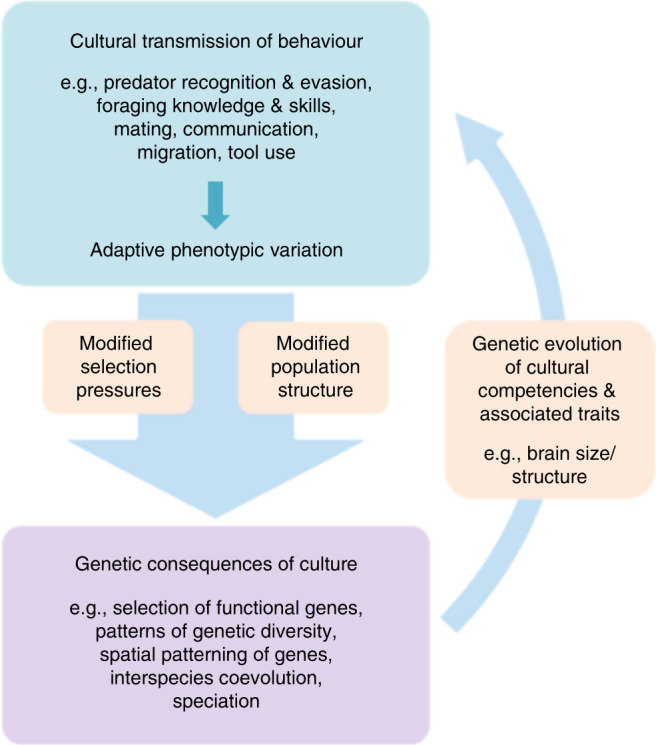
Principal interacting processes of gene–culture coevolution. Many behaviours are transmitted through culture and often give rise to adaptive phenotypic variation and subsequent genetic consequences (examples of both are provided). These changes in genes also feedback on cultural transmission. Orange boxes highlight common mechanisms

Genes and culture are both inheritance systems that transmit information between organisms and generate phenotypic change (see Box 1 for definitions). Social learning is the key ingredient of culture. Animals learn important skills from each other, including what to eat and where to find it, how to recognise and escape predators, and which migratory pathways to take through their environments. These cultural adaptations affect population structures as well as the physical and social environment that elicits genetic evolution. Thus, cultural behaviour may select for particular functional genes, influence patterns of genetic diversity, and spark speciation. When cultural activity is an important determinant of fitness, it can generate selection for traits that further enhance cultural competencies, allowing genes and culture to coevolve reciprocally (Fig. 1).

Early reviews assumed that non-human cultures were insufficiently stable to affect genetic evolution^1,2^. However, recent research has established that animal culture is present in insects, birds, fishes and mammals^3,4^, that it can have important impacts on fitness (e.g., ref. ^5^), that it can be stable over many generations (e.g., refs. ^6,7^), and that it can affect evolutionary dynamics even when transient (e.g., refs. ^8,9^). Here, we review evidence from across the animal kingdom that the strength and direction of selection, as well as population genetic structure, are influenced by cultural activities in a range of species, not just humans. We define gene–culture coevolution inclusively, as occurring when cultural evolution shapes genetic evolution, often but not always entailing reciprocal interactions between the two. We compare genetics and culture as systems of inheritance, and explain the different ways in which culture modifies genetic selection. These include how culture may select for particular functional genes, whether in populations, species-wide, or in different species, and how culture shapes the structure and diversity of variation in neutral genes.

Box 1 Glossary *Conformity:* A disproportionate adherence to majority behaviour (positive frequency dependence)^2^, which may override personal adherence to an alternative.

*Cultural evolution:* Evolutionary change in behaviour, knowledge or constructed artefacts arising from social learning and transmission.

*Cultural hitchhiking:* Neutral genetic variants that happen to be carried by individuals who transmit selective cultural traits ‘hitchhike’ along the same cross-generational pathways; matrilineal cultural inheritance can hence constrain mitochondrial genetic inheritance, leading to a reduction in variance^93^.

*Cultural intelligence*: Cognitive processes supporting and/or dependent on cultural transmission.

*Cultural transmission:* Diffusion of behaviour patterns or knowledge via social learning from others’ actions or their consequences.

*Culture:* (a) broad sense—equivalent to ‘Tradition’ below^123^; (b) more elaborate sense–a communal complex of multiple traditions^56^.

*Ecotypes:* Communities within the same species exploiting different ecological niches.

*Gene–culture* (or *Culture-gene*) *coevolution:* Cultural processes shape genetic evolution by modifying the selection of genes, often entailing reciprocal interactions and feedbacks.

*Horizontal transmission:* Cultural transmission of behaviour between members of the same generation^13^.

*Phenotypic plasticity*: The ability of a genotype to produce different phenotypes in different environments.

*Social learning:* Learning that is facilitated by observation of, or interaction with, another animal or its products^4,17^.

*Tradition:* A behaviour pattern shared by members of a community that relies on socially learned and transmitted information.

*Vertical transmission:* Cultural transmission of behaviour from parent to offspring^13^.

## Evolutionarily relevant properties of culture

Parallels between genetic and cultural inheritance are well-established, but cultural transmission is mechanistically different from genetic transmission and cannot simply be treated as ‘another class of gene’^3,10,11^. Although genes are transmitted at conception from parents to offspring, social learning occurs throughout the lifespan, from many different individuals. Culture thereby allows for the propagation of phenotypic variants among unrelated individuals, often within timespans significantly shorter than a generation (e.g., social transmission of predator recognition in minnows, *Pimphales promelas*^12^; socially learned mating preferences in grouse, *Centrocercus urophasianus*^9^). Such ‘horizontal cultures’, comprising learning among similarly aged peers, can be highly labile; conversely, ‘vertical cultures’, in which offspring learn from parents, can be very stable^13,14^. The stability of cultural transmission can be enhanced through conformity (i.e., a disproportionate tendency to adopt the most common behaviour), as in the foraging traditions of great tits, *Parus major*^15^. This stability allows cultural traits to be maintained as Nash equilibria, generating a ‘cultural inertia’ that can hinder adaptation to changing environmental conditions, and leading to population-specific traditions in behaviour (e.g., migratory pathways in reef fishes^16^). Through eliciting change in behaviour, often across an entire population, culture can transform the social environment, whereas cultural activities (e.g., foraging) also modify ecological circumstances. Culture thus encompasses a range of temporal scales, pathways of information flow and impacts on selection.

Cultural transmission has a number of additional properties that affect its role in genetic evolution. Culture provides a highly flexible means to adjust to novel conditions and modify selection. Much of social learning is reliant on phylogenetically ancient and widespread associative learning processes, such as classical and operant conditioning^17^. This kind of learning can be applied in an extremely flexible and open-ended manner, including learning from both conspecifics and heterospecifics, which means that animals are not restricted to learning only about environmental features previously encountered by the lineage (e.g., established predators or foods). Instead, animals can also learn about entirely novel stimuli or events, and devise appropriate responses to them (e.g., birds learn to evade a novel predator^18,19^). Via learning, animals can therefore generate adaptive responses to conditions without prior evolution of dedicated traits with suitable reaction norms. In addition to responding appropriately to changing features of their environments, such as the dangers presented by a novel predator, social learning can also generate opportunities for phenotypic change in the absence of any immediate environmental change or stressor (e.g., when orangutans, *Pongo pygmaeus*, proactively devise new food-processing techniques, social learning allows others to access hitherto-unexploited foods, such as palm heart^20^).

Culture typically leads to the production and propagation of adaptive behaviour. Learned behavioural innovations (the analogue of mutation) are usually not random but rather novel functional solutions tailored to new challenges or hitherto-unexploited opportunities^21^. For instance, among the most-celebrated examples of animal innovation are the invention by primates and cetaceans of new food-processing methods, and new dominance and courtship displays, which spread because of their perceived beneficial qualities and/or are associated with a rise in status or reproductive success^22,23^. Learned behavioural innovation is now extensively documented in animals^21^, and many such innovations are propagated through social learning (e.g., ref. ^24^). Animal social learning is also typically non-random and strategic, with evidence that individuals often disproportionately copy successful individuals and high-payoff behaviour^25,26^, enhancing the spread of adaptive variants (e.g., some insects and birds are known to copy the nest-site decisions of successful conspecifics and heterospecifics^27,28^). Good information, supporting fitness-enhancing behaviour, is far more likely to be propagated than bad information.

These features, most of which are particularly well-documented in vertebrates, mean that phenotypic accommodation through culture has the potential to be common, rapid and powerful. Cultures can quickly accumulate adaptive features^29^ and introduce novelty into phenotype space, generating diverse selection on genes^30^. Yet, cultural systems are not typically well-captured by standard population genetic or quantitative genetic models of trait evolution, but instead require dedicated theory^2,3,13,31,32^.

## Culture modifies the strength of selection

A prima facie confusing feature of culture is that it can both speed up and slow down genetic evolution, but these contrasting effects are now well-understood, thanks to theoretical work^2,13,33^. In stationary, or slowly changing, unimodal fitness landscapes, learning typically slows evolution by reducing phenotypic differences between genotypes^34–36^. This explains how cultural species such as humans and, possibly, bottlenose dolphins (*Tursiops* spp.) can live in extremely diverse habitats without major genetic adaptations^37^. However, learning usually accelerates evolution in dynamic environments that cannot be tracked effectively by selection of genes^2,13^, or in static multimodal fitness landscapes, where the existence of multiple optima means that populations can become trapped on suboptimal fitness peaks. In the latter case, learning smooths the fitness landscape, increasing the likelihood of a directly increasing path of fitness to the global optimum^33,36,38^ and helping genotypes to locate otherwise difficult-to-find fitness peaks^39^.

In cases where learning accelerates evolution, phenotypic change (henceforth ‘phenotypic accommodation’) precedes, and then facilitates genetic adaptation by modifying selection on genetic variation (‘genetic accommodation’)^40^. Although, as described above, social learning can buffer selection on genetic variation that would otherwise lead to genetic adaptation, this buffering is unlikely to be perfect, and hence will not preclude selection of alleles that increase the probability of producing the phenotype, or improve it further (a process known as ‘genetic assimilation’^41^). For instance, the dietary traditions of killer whales, *Orcinus orca*, have favoured population-specific genes influencing morphology and digestion^42,43^.

Social learning can also elicit the selection of genetic adaptation in other traits (genetic accommodation). For instance, mate-choice copying, where the choice of mating partner is influenced by the mate-choice decisions of other individuals, is found in fruit flies, fishes, birds and mammals. Mate-choice copying propagates mating preferences over short time periods, such as a single season, yet population genetic models have shown that it can substantially enhance the strength of sexual selection on male traits^8^. Birdsong provides another illustration of how cultural change, which is rapid at least with respect to rates of genetic change, can nonetheless be consequential for genetic evolution, influencing patterns of migration and assortative mating, and facilitating speciation^44–46^. In sum, theoretical work leads to the expectation that genetic accommodation and genetic assimilation in response to culture could be widespread in animal populations, but this has been little investigated. Further examples of how cultural plasticity can precede and facilitate genetic evolution, and affect evolutionary rates, are considered in more detail in the following section.

## Culture creates new selection pressures

### Culture changes selection on functional genes

Among the processes of gene–culture coevolution, attention has overwhelmingly focussed on relationships between the distributions of functional genes within a population and cultural variants. Correlations are expected if cultural innovations alter the selection regimes for particular genes, as individuals with the same genes can have different fitness in different cultural contexts. Given it is impossible to demonstrate the causes of past episodes of adaptive evolution experimentally, the strongest evidence comes from humans, where extensive genetic sampling can be combined with historical and archaeological data. For example, the coevolutionary relationship between dairy farming and adult lactase production is well-established^47^. More generally, diverse agricultural practices are thought to have inadvertently selected for alleles expressed in enhanced metabolism of the increased starch, carbohydrates, alcohol and so forth, found in agricultural diets^31,48^. Genomic studies have identified over 100 other variants subject to recent selection for which cultural practices are thought to be the primary source of selection, although the difficulty of demonstrating their causal role precludes certainty^48^.

In non-humans, the most-compelling evidence comes from killer whales, in which recent population-genomic studies show that functional genes associated with digestion differ between ecotypes (Box 1) in ways that appear adaptive, and show evidence of recent selection^42,43^. Genes associated with the methionine cycle, which is involved in protein synthesis, differ systematically between mammal-eating and fish-eating ecotypes, a contrast presumed to result from different patterns of dietary protein intake^42^. Although North Pacific and Antarctic mammal-eating ecotypes both had strong signatures of selection in these groups of genes, the precise locations of the variations were different in the two ecotypes, suggesting independent genetic routes to phenotypic change. Given the culturally mediated variation in inter-population variation in chimpanzee (*Pan troglodytes*) diets^49^, similar culturally initiated genetic variation in digestion may well be found in this species too.

Culturally transmitted foraging preferences might also have influenced the evolution of functional genes in great tits^15,50^. Providing food for these birds is popular in the United Kingdom and recent genomic comparisons of British great tits with those from the Netherlands, where bird feeding is less common, suggest there has been selection on genes involved in beak morphology^50^. Birds that use feeding stations have larger beaks, perhaps because these individuals are more effective at breaking open the provided seeds. Parid tits became famous for their foraging innovations when they learned to pierce open milk bottles to drink the cream in 1940’s Britain; this behaviour spread too rapidly to be the result of individual learning alone^51^. Artificial seeding of foraging behaviours in great tits has subsequently demonstrated that detecting and learning how to access bird feeders can spread horizontally and vertically through populations via social learning, enhancing individual learning^15^. It is therefore probable that social transmission of information about seed feeders increased their use quickly, and consequently altered selection pressure for beak size in great tits^50^. Furthermore, genetic differences within and between populations could affect culture reciprocally; through their positions in social foraging networks^50,52^, larger-beaked individuals may be more likely to spread knowledge of new feeding opportunities^53^.

Although genomic scans suggest large-scale culturally driven selection for genes in recent human evolution, with a few exceptions, such as the dairy farming-lactase case, further work is required to pin down particular instances^46,54^. This is also the case with non-humans where, for instance, it is necessary to exclude the theoretical possibility that ‘allele-surfing’ (i.e., increases in allele frequencies during post-bottleneck population growth) could have contributed to the patterns found in killer whales^55^.

### Culture favours genes enhancing adaptations for culture

A substantial reliance on social learning has been predicted to select for genetic variants that enhance such learning species-wide, shaping supportive neural traits such as encephalisation, energy production or plasticity, or modifying life-history traits such as a longer juvenile period available for learning or enhanced parental support. 'Cultural drive’ or ‘cultural intelligence’ hypotheses^2,37,56–62^ cite a range of evidence that cultural inheritance can enhance fitness through the development of greater competence in key behaviours such as foraging and predator avoidance^56,59^, and they propose that this in turn will enhance selection pressures for genetically coupled phenotypic traits particularly the neural and life-history variables mentioned above. These effects may in turn lead to more reliance on culture, potentially creating positive ontogenetic-evolutionary feedback loops shaping gene–culture coevolution.

Tests of the cultural intelligence hypotheses have been of two main types. Most common have been cross-species, comparative analyses, addressing the prediction that the scale of cultural inheritance in a species will be associated with selection on supportive phenotypic characters. For example, Street et al.^63^ applied phylogenetic comparative analysis techniques to published databases that span 55 primate genera and 184 species to address relationships between records of social learning and predictor variables. Evidence of greater proclivity for social learning was predicted by both measures of brain size and of reproductive lifespan. The authors concluded that results are ‘consistent with the hypothesis that the evolution of large brains, sociality, and long lifespans has promoted reliance on culture …. in turn driving further increases in brain volume, cognitive abilities, and lifespan in some primate lineages’^63^.

Culture relies not only on social learning but also on intermittent behavioural innovation, and similar comparative analyses have identified relationships between records of innovation and brain size in both primates^64^ and birds^65^. Reinforcing these correlational analyses, a recent mechanistic model of brain evolution concluded that ‘our results are consistent with aspects of various cultural hypotheses for brain evolution’^66^.

A second approach is to compare closely related species differing in cultural richness, recently explored in a comparison between orangutan species, in which a more extensive cultural repertoire has been described for the Sumatran (*Pongo abilii*) than for the Bornean species (*P. pygmaeus*)^67^. Consistent with the cultural intelligence hypothesis, the Sumatran species have brains reported to be 2–10% larger and showed superior performance in cognitive tests conducted in comparable captive environments^67^.

Evolutionary effects of culture may explain a further life-history phenomenon, the existence of menopause not only in humans but in whales, where females of matrilineal species may live long after their reproductive span^68^. Modelling studies have concluded that menopause can evolve through inclusive fitness benefits^69^. Menopause is predicted to be favoured when females’ relatedness to the group and ability to assist relatives (e.g., by providing a highly competent model from whom to learn) increase with age, but continued reproduction would reduce their capacity to assist relatives^69^. Older female killer whales are known to be repositories of such extensive adaptive knowledge^70^.

Correlational tests of the cultural intelligence hypothesis outlined above have, however, been constrained by relatively crude measures of the scope of cultural intelligence in any given species, often resting on post hoc analysis of publications that report non-standardised measures of social learning. In future, the field will benefit from the development of more comparable and systematic variables to be applied in such analyses.

### Culture generates selection across species

One difficulty with demonstrating gene–culture coevolution arises when genes and culture are both spread predominantly through transmission from parents to offspring. When culture changes how species interact, however, it can also influence genetic evolution across species boundaries, removing this potential confound. For example, experimental studies with brood parasitic cuckoos and hosts have established that culture may alter the strength of selection across time differently to when culture is absent. Knowledge of the threat of brood parasites (‘cuckoos’) is maintained in groups via social learning among naïve fairy-wren (*Malurus cyaneus*) hosts^71,72^, and reed warblers (*Acrocephalus scirpaceus*) learn about the identity of cuckoos that mimic more dangerous enemies horizontally from peers^18,19,73^. In reed warblers at least, this socially learned and transmitted information leads to increased detection of cuckoos and strengthens selection against the parasite^74^. However, common cuckoo (*Cuculus canorus*) females have a colour polymorphism, likely to be an MC1R variant^75^, that defeats host culture. Social learning about cuckoos is not generalised across morphs, so knowledge spreads quickly only about the common form. In this case, host culture generates a stronger selective advantage for cuckoos that appear different (i.e., negative frequency dependent selection) than if hosts only learned individually^18^.

Novel behaviours that spread and are maintained through culture also have the potential to initiate, or intensify, selection on interacting species. For example, recent experiments with great tits show that social transmission of foraging preferences can switch a prey species’ defence strategy from crypsis to aposematism^76^. Another candidate for interspecies gene–culture coevolution lies in the wide array of complex socially learned foraging techniques of killer whales^77^. Corkeron and Connor^78^ suggest that the seasonal migrations of baleen whales to the tropics may function to avoid killer whale predation. Although the details of baleen whale migrations seem socially learned^79^, the drive to migrate is likely to have a genetic basis that may have been influenced by the predatory cultures of killer whales.

There are many familiar examples where humans are the cultural species; these include the industrial revolution increasing selection for melanic forms of peppered moths (*Biston betularia*)^80^, and the rise of agriculture facilitating coevolution of novel pathogens with humans^81,82^. Although evidence for non-human culture altering selection in other species is at present limited, in theory this could influence any type of interaction where social learning in (at least) one party occurs. For example, previously ‘honest’ foraging bumblebees (*Bombus terrestris*) learn to rob nectar from flowers by observing others^83^—if this knowledge transmits across generations^84^, it could shape pollination efficiency and selection on the plant^83^. The onset of a new foraging culture could also rapidly shift selection on host microbiomes as they adapt to a novel resource, or influence transmission of microbial communities^85^. Culture influencing genetic change across species boundaries has great potential to demonstrate how gene–culture coevolution can operate.

## Culture shapes population genetic structure and diversity

### Culture shapes neutral genetic variation in space

When cultural knowledge strongly affects habitat use or patterns of dispersal through the social learning of habitat preferences, migration routes or foraging methods that select for different habitats, then cultural knowledge can be unevenly distributed across the range of a species. If neutral genes are being transmitted in parallel with these cultural traits—which would necessitate relatively stable vertically transmitted cultures—persistent geographical patterns of genetic variation can result. For example, in Shark Bay, Australia, foraging techniques of bottlenose dolphins (*Tursiops aduncus*) that are transmitted from mother to daughter have set up a distinctive spatial structure of mitochondrial haplotypes over scales of a few kilometres, which the dolphins could traverse in minutes. Haplotypes characteristic of a feeding type that is used in deep water map onto the greater depths, whereas in the neighbouring shallows other haplotypes predominate^86^.

Through culturally facilitated dispersal, adaptation to culturally modified environments, and the effects of cultural practices on admixture, culture has directly or indirectly shaped much of the current geography of human genes^48,87^. Similar effects may arise from culture in other species^32^. Migration routes, or navigation strategies (e.g. ‘follow the coast’), learned from parents can set up geographical patterns in neutral genetic variation at either or both ends of the route^88^. Cultural transmission of migration routes is common among birds^89^, especially long-lived species, and cetaceans, especially baleen whales; in both cases, geographical patterns of genetic variation result^79,90^. A particularly remarkable case is that of the beluga whales (*Delphinapterus leucas*), where strict matrilineal migration cultures have retained distinct mitochondrial haplotype distributions on summering grounds in eastern and western Hudson Bay, reflecting different glacial refuges of maternal ancestors, even though the whales from each side of the bay meet and mate in winter and during migration^91^. In contrast, some species, such as barnacle geese (*Branta leucopsis*) and southern right whales (*Eubalaena australis*), tend to mate within migratory cultures, leading to spatial patterns in nuclear as well as mitochondrial DNA^79,90^. Geographical effects reappear in other sections of this review, where we consider how culture may segregate populations, leading to population structure and in some cases seeding speciation, reducing diversity, or selecting for particular functional genes (Fig. 1).

### Culture can reduce genetic diversity

Culture can potentially reduce genetic diversity in socially structured populations through two related processes: cultural hitchhiking and culturally mediated migration^92^. In cultural hitchhiking, culture creates heritable variation in reproductive success or survival between different groups, so that the diversity of neutral genes transmitted in parallel with the cultural traits (i.e., vertically) is reduced to essentially those genes in the original groups with successful cultures^93^. In culturally mediated migration, culture sets up barriers or divisions within a population, inhibiting dispersal and mating. Then, the diversity of neutral as well as functional genes is reduced through processes such as bottlenecks and selection^94^. Cultural hitchhiking, as well as culturally mediated migration, require cultures that affect fitness or dispersal, respectively, that are quite stable, and that do not frequently transfer between population segments.

Both cultural hitchhiking and culturally mediated migration have been considered drivers of the very-low mitochondrial DNA diversity of five species of whale that have matrilineal social systems in which daughters typically stay grouped with their mothers while both are alive^95^. Female sperm whale (*Physeter macrocephalus*) populations are partitioned into sympatric, matrilineally based clans with distinct cultures and fitness differences, so that their exceptionally low mitochondrial diversity is consistent with cultural hitchhiking^95^. The division of killer whales into ecotypes is much deeper^77^. A strong mutual antipathy of proto-ecotypes is supported by genomic results, suggesting that a form of culturally mediated migration may be behind the killer whales’ low genetic diversity^95^.

In all these cases, it is difficult to rule out all non-cultural drivers of low genetic diversity (such as bottlenecks, population divisions, background selection or selective sweeps without cultural input), and several processes may have contributed^92^. However, it is telling that both hominins and those matrilineal whale species with particularly clear evidence that culture affects behaviour and fitness^10,37^ have remarkably low genetic diversity. Similar processes may also have affected genetic diversity in birds^92,96,97^, as described below.

### Culture may drive the early phases of speciation

For speciation there are two central requirements—the separation of an existing species into groups, and reproductive isolation in the face of secondary contact. As culture can establish behavioural differences among initial population divisions more rapidly than genetic inheritance and local adaptation^77,88,98^, the role culture plays in influencing divergent selection has attracted theoretical and empirical attention, particularly concerning traits and preferences involved in assortative mating^99^.

Several researchers have argued^100,101^ (supported by population genetic models^98,102,103^) that the influence of culture on speciation depends on whether only the trait involved in mate choice is learned socially, or whether the preference is also learned. Learned-trait models (where the preference is genetically determined but the signal is socially transmitted) indicate that if culture reduces stabilising selection against new alleles affecting learning predispositions, then genetic divergence in mating preferences will arise more quickly than when traits are genetically inherited^98,102^. However, if mating traits are learned obliquely and independently of the tutor’s fitness, then in theory selection will be inhibited as the trait is learned from both mated and unmated tutors^103^. Cultural transmission of traits may even break down incipient speciation if secondary contact occurs because learning can mask genotypic variation^103,104^; the trait can be rapidly adjusted through learning (e.g., conformity of song dialect) to match the preferences of either population. For this reason, cultural transmission is primarily thought to inhibit sympatric speciation^100^.

However, where mating signals and receiver preferences are both culturally transmitted (e.g., male songbirds learn a dialect and females learn a preference from the same tutors) culture will instead promote speciation under both allopatric^98^ and secondary contact^102^ scenarios. Learned preferences will increase the strength of divergent selection acting on the trait and prevent recombination, amplifying both cultural and genetic evolution of associated loci^102^. Biases to learning (e.g., songs and preferences are learned during a sensitive period) may restrict individuals shifting phenotypes to match local preferences and further limit gene flow^99^.

Studies of culturally transmitted song in birds have generated the best lines of evidence for the role of culture in speciation^105^, with recent work demonstrating that song learning increases rates of species diversification across clades^106^. White-crowned sparrows (*Zonotrichia leucophrys*) song dialects vary strongly over small spatial scales, with restricted gene flow between dialect groups^97^. Recently, genomic studies of neighbouring subspecies (*Z.l. nutalli* and *Z.l. pugetensis*) demonstrated that song dialects likely act as a barrier to gene flow, with the strength of song discrimination correlated with genetic distance between sites^96^. Among indigobirds (*Vidua*), which are brood parasites, cultural transmission occurs across species, as males learn the song of their host species and females imprint upon that same song^107^. Switching host species therefore creates strong isolating barriers that have led to rapid recent speciation in sympatry^108^. Finally, culture-shaping speciation may even have been captured in action with the recent establishment of an apparently isolated lineage of Galápagos finches (*Geospizinae*) derived from the hybrid mating of a cactus finch (*G. conirostris*) immigrant male with a medium ground finch (*G. fortis*)—termed the ‘Big Bird’ lineage^109^. Twenty years ago, Grant and Grant^44^ showed that Darwin’s finches acquire their song-type and preference from their father (or foster-father). Although females do not sing, they mate assortatively according to their culturally inherited song type as well as by imprinting on parental morphology; culture therefore facilitates pre-zygotic reproductive isolation by constraining female mate choice, even in the absence of genetic penalties^110^. Together with changes in beak size, in only three generations this song culture has most probably contributed to allele segregation and incipient speciation of the Big Bird lineage^109^, although experiments demonstrating this mechanism remain to be done.

Finally, by coupling changes in population structure and modified selection on functional genes, cultural transmission of foraging traits or preferences could also help facilitate genetic divergence. Killer whale population structure is strongly influenced by culturally transmitted specialisations on particular prey resources, giving rise to different killer whale ‘ecotypes’ that can be distinguished behaviourally and morphologically^77^. There are significant genetic divergences between these lineages both in mitochondrial^111^ and nuclear^42^ genomes. It is believed that the ecotypes arose from cultural niche specialisation by matrilineal groups, which later developed reproductive barriers, culturally (e.g., through learned aggression towards outgroups) and/or genetically^77^. In killer whales, culturally transmitted behaviour has therefore triggered the evolution of multiple lineages considered to be currently undergoing incipient ecological speciation^77^. Although some have suggested they should now be considered different species^111^, these ecotypes are not currently recognised as such. The genetic evidence suggests that the present day global diversity of killer whales has arisen in the last 250 kya, or ~8000 generations^42^, but the genus *Orcinus* has been present in the fossil record for up to 5 My—so why are estimates of ecotype divergence so relatively recent? The most likely explanation is that the formation of ecotype lineages is a dynamic process, with learning and cultural transmission at its heart, both driving the exploitation of new niches as they arise and leading to increased specialisation risk, resulting in lineage extirpation^112^. Thus, in killer whales, cultural transmission seemingly underlies adaptive radiation into ecotypes and then incipient species, but can also increase local extirpation risk. The social learning of foraging techniques or habitat preferences by young animals has the potential to trigger genetic radiation in a variety of taxa, but this has been little investigated beyond killer whales.

The precise nature of the interactions between individuals with alternative cultures may be key to speciation, and to other processes of gene–culture coevolution. However, such interactions, almost inevitably, are rarely observed. How conspecifics whose cultures have begun to differentiate respond to each other, for example, by conformist convergence or alternatively by avoidance and conflicts, is a topic that can benefit from complementary observations in the field and experiments.

## Conclusions and future directions

Until now, consideration of gene–culture coevolution has largely focussed on the evolution of functional genes in human populations^1,31,54^. Our review extends the reach of this discussion by suggesting that culture may change the nature of the evolutionary process in diverse ways, and for many animals. Culture provides a form of inheritance that is additional to genes and our review indicates it is far from trivial in its consequences for genetic evolution; moreover the two inheritance streams can interact to influence each other’s evolution^3,11,30,46,113^.

Human culture, so prolific and dominant across the globe, has had a dramatic impact on genetic evolution in other species^114^, a process for which we now have preliminary evidence at least, in a diversity of other species. There is good evidence for both humans and non-humans that culture can shape geographic patterns of genes, and comparative phylogenetic evidence that it has driven species-wide genetic traits, such as aspects of brains and life-histories. The cultures of killer whales offer a case study of how culture may drive the evolution of functional genes within population segments, and the ecotype-fissured killer whales offer a simpler system than human culture within which to delineate fundamentals of such processes.

Two processes of gene–culture coevolution were first considered for non-humans, with their relevance for our own species explored later. Cultural hitchhiking was conceived to explain low genetic diversity in cetaceans^93^, and was later extended to humans^94,115^. Culturally mediated speciation was a topic first introduced for birds^44^ and whales^77^, but might also apply to hominins. It is now well-established that Neanderthals, Denisovans and *Homo floresiensis* coexisted with modern humans and possessed distinct behavioural repertoires^116^. Culturally transmitted ecological specialisations may have led to radiations comparable to that of killer whales in our own evolutionary past, possibly on multiple occasions.

**Fig. 2:**
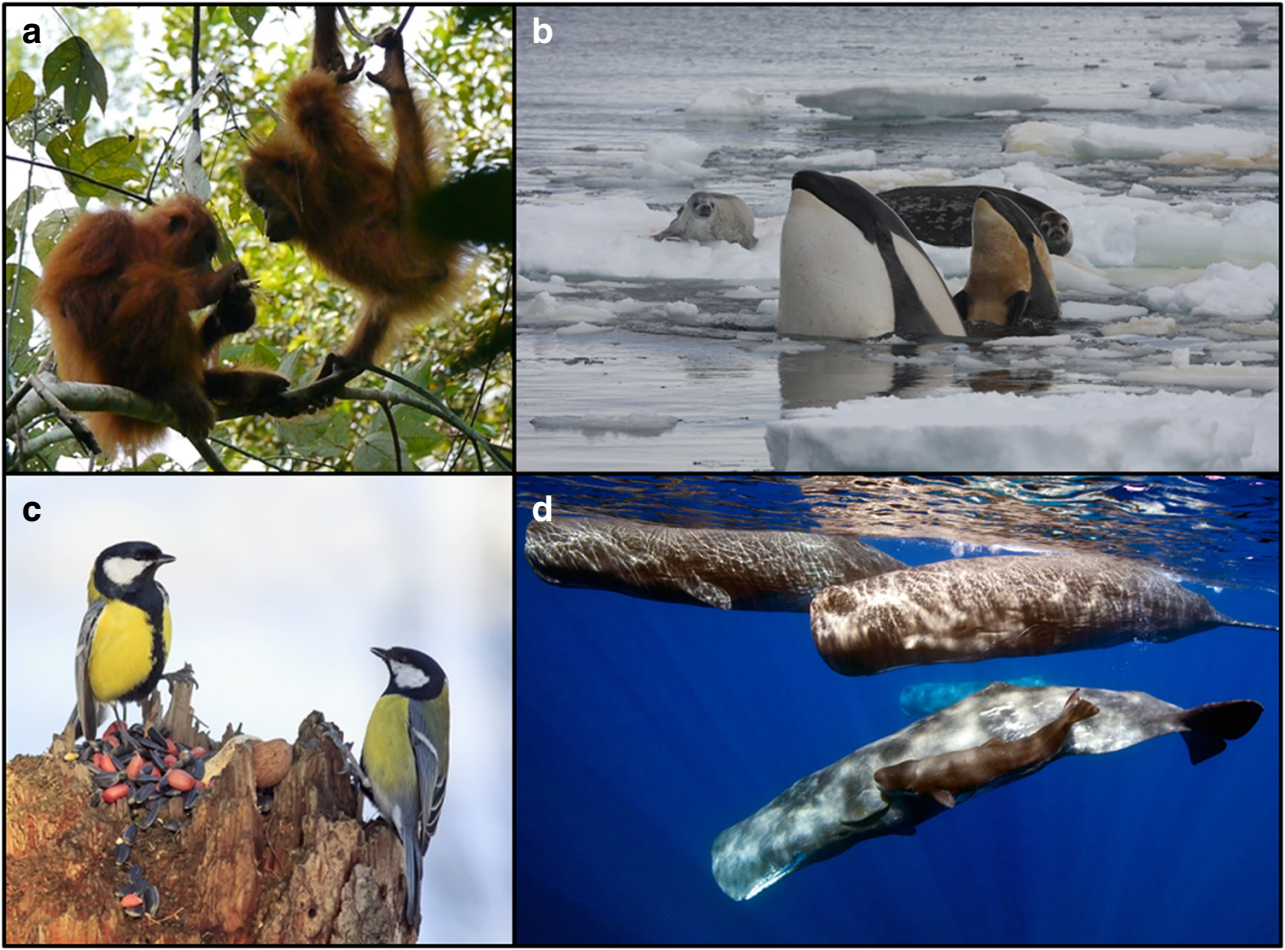
Species whose culture may have affected their genetic evolution. **a** Young orangutan peering closely at mother’s skilled tool use in foraging. Predictions that the more extensive cultural repertoire of Sumatran compared with Bornean orangutans would be associated with neuro-cognitive superiority were confirmed on the neutral ground of cognitive tests in zoos^67^. Image courtesy of Christiaan Conradie and Caroline Schuppli. **b** Young pack ice killer whale from the Antarctic assesses potential prey with mother. Members of this seal-feeding ecotype have evolved genes that assist in the digestion of mammal food^42^. Image courtesy of Robert Pitman. **c** Great tits learn foraging techniques from one another^53^. Compared with the relatively feeder-free Netherlands, great tits living in Britain where feeders are common have evolved stronger jaws that are more efficient at processing feeder food^50^. Image used under licence from Fotolia/Nataba. **d** Female sperm whales live in tight matrilineal groups whose distinctive cultural behaviours may have reduced the diversity of hitchhiking mitochondrial genes^95^. Image courtesy of Wayne Osborn

A sizeable body of experimental data, mathematical theory and comparative analyses collectively attests to the potential significance of gene–culture coevolution in animals. However, at this stage conclusions must often be regarded as tentative. This is primarily because few studies have genetic and cultural data that are directly comparable in terms of the organisms or groups of organisms sampled, or in the spatial and temporal scopes of the data. Second, our evidence comes predominantly from studies of non-human primates, birds and cetaceans, taxa for which cultural transmission has received the most attention to date^5,37,117^ (Table 1; Fig. 2) but which limits scope for generalisations. Comparing across taxonomic groups is further complicated by the differing foci on various aspects of culture, such as song for birds, foraging for primates, and mate-copying for insects. It would be helpful if more systematic and comparable measures were adopted to capture cultural content (foraging, navigation, song etc.), social transmission mechanisms (copying, teaching, etc.) and the distribution patterns of cultural variants as they evolve regionally and over generations. Ideally, future studies would systematically assemble coupled data on both genomics and the diverse dimensions of cultural complexity to facilitate comparative analyses of the gene–culture coevolutionary hypotheses that we have outlined above.

**Table 1:** Processes and cases of gene–culture coevolution

Process of gene–culture coevolution	Typical or relevant characteristics of culture (not necessarily sufficient)	Stronger evidence	Weaker evidence	Circumstantial evidence
Culture selects functional genes	Transgenerational transmission of phenotypes typically stable; strong selection derives from behavioural homogeneity; conformity.	Humans (e.g., *LCT*, *AMY1*, *HbS*…)^31,48^ Killer whales (e.g., methionine-expressed alleles, …)^42^	Birds^50^	Non-human primates^a^
Culture favours adaptations for culture	Complex and diverse learning environment created by culture; adaptive advantages of strategic and high-fidelity copying; socially transmitted technology; conformity.	Humans^2,48,54,66,124^	Cetaceans^37^ Non-human primates^59,61,63,64^ Birds^46^	
Culture generates selection across species	Transgenerational transmission of phenotypes modifies selection on other species.	Humans^48,80,114^	Birds^18,74,76^	Killer whales^a^
Culture shapes neutral genetic variation in space	Stable, transgenerational transmission of phenotypes.	Humans^48,125^ Long-lived birds^90^ Beluga whales^91^ Baleen whales^79^ Bottlenose dolphins^86^		
Culture can reduce genetic diversity	Stable, transgenerational transmission of phenotypes; behavioural homogeneity.		Sperm whales^95^ Killer whales^95^ Humans^48,94^	Pilot whales^95^ False killer whales^95^ Birds^92^
Culture may drive the early phases of speciation	Stable, transgenerational transmission of phenotypes; behavioural homogeneity; conformity.	Birds^44,48,98,126^ Killer whales^77^ Humans^48^		Hominins^a^

Cases are designated as ‘stronger evidence’ where there exists compelling experimental, theoretical or correlational data that imply gene–culture coevolution. Cases with ‘weaker evidence’ are those where experimental, theoretical or correlational data are consistent with gene–culture coevolution but where plausible alternative explanations have not been ruled out. Cases described as ‘circumstantial’ are those in which gene–culture coevolution has been proposed but not yet investigated

^a^Proposal made in the text

Another limitation of the current state of knowledge is that a large portion of available evidence is correlational. Experimental studies would add clarity, but many of the best-studied taxa tend not to be well suited for manipulative experiments. Birds are an exception^15^, but the extensive evidence now accruing for social learning in insects^118^ makes this taxonomic group a promising area for development of the field^119^. For example, a recent study of a parasitoid wasp species (*Lariophagus distinguendus*) shows that early learning of host preferences has facilitated the evolution of two isolated, potentially speciating, lineages^120^, and a recent finding that fruit flies (*Drosophila melanogaster*) can carry culturally transmitted mate preferences across multiple cultural generations^121^ suggests great potential for experimental evolution of both culture and genes^122^. The study of gene–culture coevolution beyond humans may be in its infancy, but sufficient evidence now exists to see it as an exciting and significant avenue for further focused empirical research.
